# Discriminatory Climate and School Adjustment in Ethnically Minoritized Adolescents and Majority Adolescents: An Investigation of the Mediating Role of Teaching Quality

**DOI:** 10.1007/s10964-025-02147-2

**Published:** 2025-02-07

**Authors:** Birgit Heppt, Miriam Schwarzenthal, Jan Scharf

**Affiliations:** 1 TU Dortmund University, Dortmund, Germany; 2 Institute for Educational Quality Improvement (IQB) at Humboldt-Universität zu Berlin, Berlin, Germany; 3 University of Wuppertal, Wuppertal, Germany; 4 DIPF | Leibniz Institute for Research and Information in Education, Frankfurt, Germany

**Keywords:** Discriminatory climate, Teaching quality, Reading comprehension, Reading motivation, School belonging

## Abstract

Discriminatory teacher beliefs and behaviors, as reflected in a discriminatory climate, are negatively related to student adjustment, but little is known about the classroom processes contributing to this relationship. This study investigated the role of teaching quality as a mechanism behind the associations between a discriminatory climate at school and students’ school adjustment. The study used PISA data collected in Germany in 2018 (*N* = 2947; *M*_age_ = 15.47 years, *SD* = 0.65; 48.4% girls) and included ninth graders (1) from ethnically minoritized groups that are highly stigmatized (i.e., with heritage from Turkey, the SWANA region, sub-Saharan Africa, and Kurdish areas; *n* = 198), (2) from other ethnically minoritized groups (*n* = 445), and (3) from the ethnic majority (*n* = 2304). The students in Group 1 reported a more discriminatory climate at school than the other student groups did. Multilevel analyses revealed that a discriminatory climate was negatively related to all three indicators of school adjustment (i.e., reading comprehension, reading motivation, and school belonging). Adolescents who perceived a stronger discriminatory climate experienced lessons as less structured and more disruptive, highlighting the mediating role of classroom management in the relationship between discriminatory climate and adolescents’ school adjustment. Thus, a discriminatory climate at school hampers adolescents’ educational outcomes not only directly, but also via teachers’ instructional behavior in class.

## Introduction

With ongoing migratory movements, classrooms in many countries, including Germany, are characterized by increasingly diverse student populations. Although schools and teachers are called on to ensure equal educational opportunities for all students (e.g., OECD, 2018), students from ethnically minoritized groups continuously show lower academic achievement than their ethnic majority peers do both internationally (OECD, 2023) and in Germany (e.g., Henschel et al., 2019). These educational disparities are often attributed to student- and family-related conditions, such as differences in socioeconomic status (SES) or language skills (e.g., Henschel et al., 2019; OECD, 2023). However, discrimination by teachers (e.g., the endorsement of stereotypes toward ethnically minoritized groups) and associated classroom processes, possibly mediating, aggravating, or attenuating its effects, also need to be considered in explaining educational inequalities. Personal discrimination, which refers to minoritized students’ individual experiences of ethnic-racial discrimination by teachers, has repeatedly been shown to be negatively associated with the school adjustment of ethnically minoritized students (for a recent meta-analysis, see Civitillo et al., 2023). However, less is known about the effects of a discriminatory climate, comprising students’ perceptions of discriminatory teacher beliefs and behaviors. Such perceptions are formed not only by personal discriminatory experiences but also by witnessing discrimination, suggesting that they may affect both minoritized students and majority students. Whereas the emerging literature indeed suggests negative relationships between a discriminatory climate and student achievement and engagement in minoritized and majority students (Baysu et al., 2023; Del Toro et al., 2024), the classroom processes contributing to this relationship remain largely unclear (Denessen et al., 2022). Building on the pivotal role of instructional quality in fostering student learning and socioemotional adjustment, the present study focused on (student-perceived) teacher behavior in the classroom as a potential mediator of the relationship between a discriminatory climate (comprising perceptions of teacher discriminatory beliefs and behavior) and school adjustment.

As ethnically minoritized students in Germany are a tremendously diverse group and probably face different degrees of discrimination (and therefore stigmatization) in German society, this study further examined whether the effects differ on the basis of ethnic group membership. Using data from the Programme for International Student Assessment (PISA) collected in Germany in 2018, the study focused on secondary school students. Teacher‒student relationships are highly influential for student adjustment during adolescence (Emslander et al., 2024). Moreover, secondary school students have been shown to be particularly sensitive to school (dis)engagement (Wang & Eccles, 2012), as reflected in students’ cognitive (e.g., reading comprehension), motivational (e.g., reading motivation), and socioemotional (e.g., sense of school belonging) outcomes. Considering both the instructional quality and the ethnic composition of the student body in the classroom, this study allows for investigating both mediating and moderating effects in the interplay of a discriminatory climate and school adjustment.

### Discriminatory Climate at School and Membership in Ethnically Minoritized Groups

Some teachers have stereotypes and (sometimes unintentionally) promote discrimination and unequal treatment of students on the basis of their cultural or ethnic affiliations. Teachers, on average, have more negative implicit attitudes toward students from ethnically minoritized groups than toward those from ethnic majority groups (for a meta-analysis, see Pit-ten Cate & Glock, 2019). A possible reason for these differential attitudes may be the inherently hierarchical relationship between students and teachers, accompanied by a demographic and sociocultural mismatch, with most teachers belonging to ethnic majority groups and an increasing number of students belonging to ethnically minoritized groups (Civitillo et al., 2023).

Ethnically minoritized students, who are members of groups that are more likely to be devalued and discriminated against by teachers, typically perceive a stronger discriminatory climate at school than do ethnic majority students, who merely witness discrimination but are not directly targeted by it. For example, ethnically minoritized students in Germany tend to perceive a stronger climate of unequal treatment (by teachers and students) than do ethnic majority students (Schwarzenthal et al., 2018). Similarly, across OECD countries, students from ethnically minoritized groups (vs. students from ethnic majority groups) are more likely to perceive a discriminatory school climate, marked by teachers’ misconceptions about cultural groups or pejorative comments about people from some cultural groups (Baysu et al., 2023).

In many studies, particularly those conducted in continental Europe, researchers tend to operationalize students’ membership in ethnically minoritized groups on the basis of information about their immigrant descent. For example, students are grouped as “ethnically minoritized students” if they are first- or second-generation immigrants or speak another language at home than the language of instruction (Baysu et al., 2023). However, the group of people of immigrant descent is extremely heterogeneous with respect to cultural and ethnic affiliations. Merely relying on this broad, overarching category may mask differential experiences and perceptions by students from different ethnic groups (Vietze et al., 2023). Research from the U.S., for example, revealed that monoracial (Black) students perceive a stronger stereotyping climate at school than students of other races do (Byrd, 2014).

Considering students’ membership in a more or less stigmatized ethnic group in German society may therefore provide more nuanced insights into the relationship between minoritized student status and perceptions of a discriminatory school climate. Recently, an increasing number of studies has demonstrated differential experiences of personal discrimination across specific ethnic group memberships in Germany. These studies revealed that people of Turkish or Arabic descent tend to report particularly high levels of personal discrimination (Schotte et al., 2024; Vietze et al., 2023). As people of Turkish or Arabic descent are often perceived as Muslim, these results may be partly explained by societal Islamophobia, which is a highly salient form of discrimination in Germany (e.g., Kaddor et al., 2018). In addition, recent studies have demonstrated that experiences of personal discrimination vary on the basis of participants’ skin color and that Black Germans experience particularly high levels of discrimination (Aikins et al., 2021; DeZIM, 2023). To date, however, there is little knowledge about differential perceptions of a discriminatory school climate among more or less stigmatized ethnic groups in Germany.

### Discriminatory Climate at School and School Adjustment

Teacher attitudes, perceptions, and actions play crucial roles in shaping educational inequalities by ethnic origin (Denessen et al., 2022; Turetsky et al., 2021). A recent meta-analysis of 69 studies revealed small to moderate negative associations between personally experienced ethnic-racial discrimination by teachers and ethnically minoritized students’ GPA and test scores, school motivation, and school belonging (Civitillo et al., 2023). Importantly, only three of the studies included in the meta-analysis were from Germany. Whereas age did not moderate the associations between ethnic-racial discrimination by teachers and student well-being or academic outcomes (Civitillo et al., 2023), previous meta-analyses revealed that the impact of discrimination on school adjustment was greatest in middle adolescence (vs. early or late adolescence; Benner et al., 2018).

Extensive research has thus demonstrated the negative effects of personal discrimination experiences on ethnically minoritized students’ adjustment. More recently, attention has shifted toward examining the impact of a discriminatory school climate on all students. For ethnically minoritized students, a discriminatory climate communicates that their group is not respected in the school context. At the same time, even ethnic majority students, who are typically not targeted directly by discrimination, can be negatively affected by a discriminatory climate, as it implies the morality of the larger organization which may lead to distress and withdrawal (Jaurique et al., 2019). Along these lines, stronger perceptions of a discriminatory climate at school were related to lower scores on standardized tests for math and reading for both ethnically minoritized and ethnic majority adolescents from 60 countries (Baysu et al., 2023). In this study, ethnically minoritized students experienced the climate as more discriminatory than did ethnic majority students but the effects on student outcomes were comparable among both groups. In addition, in math classrooms with a high prevalence of teacher ethnic-racial discrimination, adolescents with diverse ethnic-racial identities have lower math course grades and test scores, and show reduced classroom engagement (Del Toro et al., 2024). In the German context, perceptions of an unequal treatment in the classroom were stronger among ethnically minoritized groups than among the ethnic majority and were related to more behavioral school disengagement and worse grades among both groups of adolescents (Schachner et al., 2021).

### Teaching Quality as a Mediator of the Relationship between Discriminatory Climate at School and Adolescents’ School Adjustment

Thus, there is growing evidence that ethnically minoritized students perceive greater levels of (personal) discrimination and a stronger discriminatory climate than their counterparts do. Perceptions of a discriminatory climate, in turn, are related to more negative school adjustment among both ethnically minoritized and ethnic minority students (Baysu et al., 2023; Del Toro et al., 2024). However, the classroom processes explaining this relationship have rarely been studied. Building on theoretical models of student learning opportunities, it can reasonably be assumed that teachers’ attitudes and stereotypes guide their perceptions and actions—as reflected in students’ perceptions of a discriminatory classroom climate—and, therefore, have an impact on teacher‒student interactions in the classroom (e.g., Denessen et al., 2022). These teacher‒student interactions, in turn, play important roles in student outcomes such as school belonging (e.g., Allen et al., 2021), motivation, and achievement (e.g., Kunter et al., 2013).

Researchers typically agree that high-quality interactions can be described by three generic dimensions of teaching quality, namely, classroom management, individual student support, and cognitive activation (e.g., Praetorius et al., 2018). Classroom management refers to structural elements of classroom teaching, such as the establishment of a set of clear rules and the effective handling of disturbances. Successful classroom management should thus contribute to the efficient use of study time, which is considered important for student learning and achievement (e.g., Seidel & Shavelson, 2007). Moreover, clearly structured and well-organized lessons may increase students’ learning motivation (Rakoczy et al., 2007). Individual student support describes the degree to which the classroom climate is perceived as positive, encouraging, and respectful, with teachers providing constructive feedback, using errors as learning opportunities, and adapting their instructional behavior to their students’ individual needs. In terms of the social and emotional aspects of student‒teacher interactions, individual student support is believed to be an important basis for student motivation and socioemotional adjustment, such as well-being and school adjustment (for an overview, see Praetorius et al., 2018). Finally, cognitive activation comprises teaching strategies that aim to foster students’ active engagement with the lesson content, for example, by providing students with challenging tasks and activating prior knowledge (e.g., Praetorius et al., 2018). Such teaching behavior should help students gain deeper conceptual understanding and, thus, be particularly beneficial for student learning and achievement (e.g., Kunter et al., 2013).

Previous research largely supports the notion that classroom management, individual student support, and cognitive activation are important preconditions for students’ school adjustment (for a meta-analysis, see Wang et al., 2020). Although not completely unequivocal, the extant literature points to differential relationships between the three dimensions of teaching quality and different indicators of students’ school adjustment (for an overview, see Praetorius et al., 2018). While both classroom management and cognitive activation have been identified as predictors of students’ learning gains (e.g., Kunter et al., 2013), individual student support has been shown to have a positive relationship with students’ socioemotional outcomes (e.g., Ruzek et al., 2022). Notably, some studies have also reported positive associations between individual student support and student achievement, possibly reflecting aspects of differentiation and adaptive support, which are also sometimes subsumed within this dimension of teaching quality (Praetorius et al., 2018). Whereas both classroom management and individual student support are considered positively linked with student motivation, these assumptions have only partly been supported by prior research (Praetorius et al., 2018).

In summary, previous research highlights that discrimination—with stereotyping as its antecedent—as well as teaching quality are important predictors of students’ school adjustment. However, the interplay of these variables in explaining student outcomes has rarely been investigated. As an exception, a recent longitudinal study revealed that the association between ethnic-racial discrimination and (lowered) math achievement was mediated by student perceptions of the math classroom climate (Del Toro et al., 2024). Importantly, to assess math classroom climate, the authors combined student perceptions of core aspects of teaching quality, such as student support and classroom management, into a global indicator. As teachers’ attitudes, including their biases and discriminatory assumptions (Denessen et al., 2022), guide their practices and social interactions in the classroom, it can be assumed that teachers who create a discriminatory climate, for instance, by devaluating members of certain ethnically minoritized groups or expressing lower expectations toward them, provide less efficient, activating, and supportive classroom teaching. Building on the large body of research on teacher expectancy effects (Wang et al., 2018), it seems plausible that teachers who have lower expectations toward minoritized groups, create less stimulating learning environments for these students by delivering less challenging tasks or providing less helpful feedback (Gentrup et al., 2020). Similarly, it can be assumed that it may be more difficult for teachers to build positive and respectful student–teacher relationships, provide adequate support, and deliver clearly structured and undisturbed lessons if they are perceived as discriminatory. Such effects may be more pronounced—and more easily perceivable for all—in classrooms with a greater share of ethnically minoritized students.

### The Role of School Ethnic Composition as a Moderator of the Relationship between Discriminatory Climate and Adolescents’ School Adjustment

Overall, school composition is considered a crucial contextual condition for adolescents’ school adjustment and for teachers’ classroom behavior, as the student body affects, for example, school bonding and teaching practices via peer interactions and expectations (Thrupp et al., 2002). However, ethnic composition is particularly important. With respect to the association between a discriminatory school climate and school adjustment, it is expected that the share of ethnically minoritized students has a moderating role. In the presence of a more heterogeneous student population, ethnically minoritized students may perceive less discrimination by peers (Vervoort et al., 2010) and teachers (Civitillo et al., 2023). The underlying assumption is that ethnic discrimination is a consequence of an imbalance of power in the social school context, which might be challenged by greater diversity (D’hondt et al., 2021; Juvonen et al., 2018) and support among ethnically minoritized students (Civitillo et al., 2023). In addition, with respect to the school adjustment of ethnically minoritized students, greater ethnic diversity at school is expected to have a positive effect on an individual’s perceived acceptance at school, that is, on their school belonging (Johnson et al., 2001).

Empirical studies on the perceived personal discrimination and school adjustment of ethnically minoritized students typically analyze the proportion of peers from ethnically minoritized groups (Baysu et al., 2024). However, with respect to discrimination in school, the empirical evidence is mixed (Baysu et al., 2024). For the secondary school context in Belgium, for instance, one study showed that perceived discrimination by peers and teachers was not related to ethnic composition (D’hondt et al., 2021). By contrast, a more recent study that investigated trajectory patterns of perceived discrimination of students of Turkish and Moroccan backgrounds revealed that students experiencing lower discrimination over time attended schools with a greater share of ethnically minoritized students (Baysu et al., 2024). Other studies analyzed composition effects on students’ outcomes. A study in Belgian secondary schools reported a greater sense of school belonging for ethnically minoritized students in classrooms with a greater percentage of peers of coethnic descent (Demanet et al., 2016). In the German context, empirical findings indicate greater intrinsic motivation to learn among ninth graders in classrooms with a greater proportion of language minority students (Rjosk et al., 2015).

Considering both perceived discrimination and adjustment in school, the abovementioned longitudinal study in Belgian secondary schools demonstrated that adolescents in schools with a greater share of ethnically minoritized groups perceived less personal discrimination over time with positive effects on their school adjustment (Baysu et al., 2024). In a similar vein, but analyzing specifically the moderating role of school ethnic composition, meta-analytic findings indicated a protective effect of a greater share of students from minoritized ethnic groups on the negative relationship between discrimination by teachers and students’ school adjustment (Civitillo et al., 2023). Accordingly, a greater proportion of ethnically minoritized students seems to compensate for the negative effects of a discriminatory climate on the academic outcomes of these students.

## Current Study

While educational research has shown that a discriminatory climate can hamper the school adjustment of both ethnically minoritized and ethnic majority students, the contextual mechanisms in the classroom behind this negative association are still largely unknown. Consequently, the aim of this preregistered study was to investigate *how* teaching practices and interactions between teachers and students may explain the relationship between a discriminatory climate and students’ school adjustment (access to preregistration via https://osf.io/rs64u/). It is hypothesized that students who belong to groups experiencing different degrees of discrimination in society perceive the discriminatory climate differently. Specifically, it is assumed that students who belong to groups experiencing higher levels of discrimination in German society—students from Turkey, the SWANA region^1^, sub-Saharan countries, and Kurdish areas—report a more discriminatory climate than those who belong to groups experiencing lower levels of discrimination, that is students from the ethnic majority and from other ethnically minoritized groups (Hypothesis 1). As students’ perceptions of a discriminatory climate are negatively related to their school adjustment, it is further assumed that students who perceive a more discriminatory climate show poorer school adjustment, as indicated by their reading achievement, reading motivation, and sense of school belonging, after controlling for important confounding variables (Hypothesis 2). Although previous research has suggested that the effects of a discriminatory climate are similar for both ethnically minoritized and ethnic majority students, this study investigates interactions between minoritized student status and discriminatory climate exploratorily. The negative relationship between a discriminatory climate and students’ school adjustment should, in turn, be mediated by teaching quality, with a greater perceived discriminatory climate being related to lower teaching quality and lower teaching quality being related to poorer school adjustment. In particular, cognitive activation and classroom management are expected to be related to students’ academic achievement (i.e., reading comprehension) and individual learning support is expected to be related to students’ sense of school belonging (Hypothesis 3). Whereas both classroom management and individual student support have been suggested to be positively linked with motivational outcomes, prior research has not provided strong support for these assumptions. Therefore, the relationship between teaching quality and reading motivation is investigated in an exploratory manner. Finally, the negative relationship between a discriminatory climate and students’ school adjustment is expected to be weakened in the presence of a greater proportion of ethnically minoritized students, thus considering the proportion of ethnically minoritized students as a moderating variable (Hypothesis 4).

## Methods

### Study Design and Sample Selection

This study used data from the PISA that were collected in Germany in 2018 (Mang et al., 2021a). Data were made available by the Research Data Centre at the Institute for Educational Quality Improvement (FDZ at IQB). The PISA is an international large-scale assessment that focuses on 15-year-olds and regularly assesses their achievement in reading, math, and natural sciences. In Germany, different types of schools from all 16 federal states are randomly selected for participation, followed by the random selection of 15-year-old students within the schools. These 15-year-olds typically attend different classrooms and grade levels (Grades 7 through 13 in PISA 2018 in Germany). In addition to this regular sample of 15-year-olds, a supplementary German sample consisting of 15 randomly selected Grade 9 students per school is recruited (Mang et al., 2021b). This supplementary sample allows for investigating relationships within the classroom context and includes students from different age groups (ages 13 through 19 years in PISA 2018 in Germany). For this supplementary sample, students are drawn from *a single* Grade 9 classroom in general schools and from *all* Grade 9 classrooms in special schools (15-year-old students who had already been selected for participation in the regular sample, could not be drawn for participation in the sample of Grade 9 students). The sampling procedures allow for full representativity for both student populations (for a detailed description of the study design and the sampling procedure, see Mang et al., 2019).

Given the study’s focus on classroom-level effects, analyses are based on this supplementary German sample of Grade 9 students (*N* = 3567 students from 225 schools). Students who did not provide the information necessary for determining their ethnic origin were excluded from the analyses. Moreover, owing to the somewhat different sampling procedures at special schools compared with those at all other types of schools, students attending special schools were not included in the analyses. The analysis sample thus consisted of 2947 students from 201 classrooms. More than one-third of the students (38.1%) attended academic track schools (i.e., leading to a university entry certificate), whereas the majority (61.9%) attended nonacademic track schools (i.e., preparing for vocational training). The students were, on average, 15.47 years old (*SD* = 0.65, *min* = 13, *max* = 19); 48.4% of them identified as girls, and 51.6% identified as boys, whereas nonbinary was not an option in the questionnaire.

#### Ethnically minoritized groups

Information on the students’ own country of birth and their parents’ country of birth was used to determine their status as ethnically minoritized students vs. ethnic majority students. In determining the grouping of students as either ethnically minoritized students or ethnic majority students, the study followed recent developments in the political debate in Germany (German expert commission on the framework conditions for integration capability, 2021) and in national (Henschel, Heppt, et al., 2023) and international large-scale assessment studies, including the PISA (Mang et al., 2021b). In line with these suggestions, students who had either immigrated to Germany themselves (first generation) or whose parents had both immigrated to Germany (second generation) were considered students of ethnically minoritized groups. Students with one foreign-born parent or with both parents born in Germany were classified as ethnic majority students (*n* = 2304). The major reason for not classifying students with one foreign-born parent as members of an ethnically minoritized group is to acknowledge that their socialization, including socioeconomic and educational family background, as well as their educational success and achievement in the German school system, more closely resemble those of students with both parents born in Germany than those of first- or second-generation immigrant students (Henschel et al., 2019; Will, 2019). Given the study’s interest in differential effects for minoritized students who are likely to perceive greater levels of discrimination in German society, minoritized students with heritage from Turkey, the SWANA region, sub-Saharan Africa, and Kurdish areas were classified as “minoritized youth from stigmatized ethnic groups” (*n* = 198)^2^. All other first- or second-generation immigrant students formed the “students from other ethnically minoritized groups” (*n* = 445).^3^ Students who could not be assigned to any of the two ethnically minoritized groups unequivocally (*n* = 37) were excluded from further analyses.

### Measures

The PISA dataset provides standardized person parameters for all the variables. Plausible values (PVs; *M* = 500, *SD* = 100) are provided for achievement tests, such as the reading comprehension test, and weighted maximum likelihood estimates (WLEs; *M* = 0, *SD* = 1) serve as person parameters for the scale scores (e.g., intrinsic reading motivation, sense of school belonging). Both PVs and WLEs are derived from a scaling procedure within an item response theory (IRT) framework (Embretson & Reise, 2000), which assumes a probabilistic relationship between a person’s item responses and the underlying latent construct. The scaling of the achievement tests (e.g., reading comprehension) integrates IRT and latent regression modeling. First, considering important background variables that are associated with the underlying latent proficiency, a population model for the proficiency distribution was estimated. Second, individual probability distributions were modeled for each student and ten PVs per student were randomly drawn from this posterior distribution, thus accounting for potential uncertainty in the data. WLEs, like PVs, are estimates of the latent underlying constructs, but their estimation does not involve latent regression modeling (for a detailed description of the scaling procedure, see Mang et al., 2019; OECD, 2020). While these standardized parameters were used for all inferential analyses, descriptive statistics of the scale scores were based on mean scores, as these allow for an easier interpretation. Reading was the major domain in the assessment cycle in 2018, and core variables such as students’ perceptions of teaching quality were only assessed for instruction in German language but not for math and natural science classes. Therefore, competence scores in these latter two domains were not considered in the present analyses.

#### Discriminatory climate

To assess the discriminatory climate at school, the students answered four items asking them about the number of teachers at their school who had biases against ethnically minoritized groups (e.g., “Teachers in school say negative things about people from some cultural groups”). The scale was developed within the PISA framework (OECD, 2020), considering prior work on the assessment of ethnic-racial discrimination at school (Fisher et al., 2000; Wong et al., 2003). All the items were answered on a 4-point Likert scale ranging from 1 (*none or almost none of them*) to 4 (*all or almost all of them*), and the internal consistency was good (α = 0.85, ω = 0.85). A factor analysis (principal component analysis [PCA] with oblimin rotation) revealed a one-dimensional structure of the construct with an eigenvalue = 2.76, factor loadings λ ranging from 0.75 to 0.88, and a share of explained variance of 69.0%.

#### Reading comprehension

Adopting a broad and functional perspective of reading literacy, the PISA 2018 reading framework considers proficient readers to be able to understand, use, evaluate, reflect on, and engage with different kinds of texts (i.e., continuous texts such as newspaper reports and letters and noncontinuous texts such as tables and diagrams) that may be presented in a printed or screen-based form (OECD, 2019). Students work on a range of different reading tasks, requiring them to locate relevant information in the texts, assess the quality and credibility of different text sources, and draw inferences. In total, the PISA 2018 reading assessment comprised 245 items in multiple-choice and constructed-response formats. In this study, tasks were administered in a computer-based and multistage adaptive assessment framework, providing each student with only a small subset of tasks that best fit their ability (i.e., multi-matrix design; Gonzaléz Rodríguez & Rutkowski, 2010). The reading assessment was scheduled to last 60 min per student (Mang et al., 2019). For each student, ten PVs were provided as indicators of their reading proficiency.

#### Intrinsic reading motivation

Five items, scored via a 4-point Likert scale ranging from 1 (*strongly disagree*) to 4 (*strongly agree*), were used for assessing students’ intrinsic reading motivation (e.g., “Reading is one of my favorite hobbies”). The three inverse items were recoded prior to the analyses. The internal consistency of the scale was good (α = 0.88, ω = 0.88), and PCA suggested a one-dimensional factor structure of the construct (eigenvalue = 3.39, factor loadings of 0.77 ≥ λ ≥ 0.87, explained variance of 67.9%).

#### Sense of school belonging

The measure of students’ sense of school belonging consisted of six items (e.g., “I feel like I belong at school”), which were answered on a 4-point Likert scale (1 = *strongly agree*, 4 = *strongly disagree*). The answers were recoded prior to the analyses so that higher values indicated a stronger sense of belonging. The internal consistency of the scale was good (α = 0.80, ω = 0.80) and the construct can be considered one-dimensional (eigenvalue = 3.04, factor loadings of 0.65 ≥ λ ≥ 0.78, explained variance of 50.7%).

#### Mediating variable: Student-reported teaching quality during instruction in the school subject German

There were three scales for assessing the three basic dimensions of teaching quality, namely, classroom management, individual student support, and cognitive activation, as perceived by the students. All items referred to instruction in the school subject German. The classroom management scale consisted of five items (e.g., “There is noise and disorder”); the individual student support (e.g., “The teacher gives extra help when students need it”) and cognitive activation (“The teacher sets clear goals for our learning”) scales each contained four items. For each statement, the students had to indicate how often a certain behavior occurred in their German classes via a 4-point Likert scale ranging from 1 (*in all lessons*) to 4 (*never or almost never*). The answers of the three scales were recoded so that higher values indicated higher teaching quality. While the internal consistency was good for the classroom management (α = 0.86, ω = 0.86) and individual student support scales (α = 0.87, ω = 0.87), it was only satisfactory for the cognitive activation scale (α = 0.73, ω = 0.73). Performing a PCA with the items of all three scales resulted in a three-dimensional structure (three eigenvalues > 1, ranging from 1.11 to 4.40, and an overall share of explained variance of 63.7%) with high factor loadings on the intended factor (classroom management: 0.73 ≥ λ ≥ 0.84, individual student support: 0.78 ≥ λ ≥ 0.89, cognitive activation: 0.54 ≥ λ ≥ 0.81) and only small cross-loadings of cognitive activation on classroom management (−0.11 ≥ λ ≥ 0.27). The three dimensions of teaching quality can, thus, be considered three factors.

#### Proportion of minoritized students in the classroom

The proportion of students from ethnically minoritized groups in the classroom was determined by aggregating individual student information on whether they formed part of the ethnic majority or minoritized groups at the classroom level. This aggregated variable ranged from 0 to 1 and higher values indicated a greater share of minoritized students in the classroom.^4^

#### Control variables

This study controlled for numerous variables that have previously been found to relate to (perceived) discrimination or discriminatory climate and to one or several of the dependent variables (i.e., reading comprehension, intrinsic reading motivation, and sense of school belonging). Control variables at the individual level were students’ age, gender (0 = female, 1 = male), language spoken at home (0 = only or mostly German, 1 = mostly another language), and family SES. The indicator of the SES was the Economic, Social and Cultural Status (ESCS) index, as provided in the PISA dataset. The ESCS index is a standardized composite score that incorporates the highest occupational status of both parents, their highest educational qualifications, and their home possessions (OECD, 2020)^5^. Control variables at the classroom level were average SES, the school type (0 = nonacademic track, 1 = academic track), and the proportion of minoritized students in the classroom.

#### Analytical procedure and statistical analyses

In the analysis sample, the amount of missing data was negligible for most study variables, ranging from 0 (e.g., for students’ minoritized student status and reading comprehension) to 0.02% (for students’ reading motivation), but was substantially greater for sense of school belonging (17%) and discriminatory climate (45%). The scales for the latter two variables formed part of a student questionnaire that was only administered to a subsample of students and scores were thus missing by design. Analyses of the missing data revealed that the full questionnaire was more frequently administered to students on the academic track than to students on nonacademic tracks. Students with versus without missing values on the scale for sense of belonging and discriminatory climate differed with respect to their reading comprehension, SES, and perceived cognitive activation, for example. Hence, data were not missing completely at random. These missing data were handled by applying multiple imputation via the R package MICE (van Buuren & Groothuis-Oudshoorn, 2011). Along with the study variables, the imputation model included various auxiliary variables that were substantially correlated with the study variables (*r* ≥ 0.20; e.g., reading self-concept, self-reported intercultural competence, attitudes toward immigrants), thus increasing the likelihood of a missing at random mechanism (van Ginkel et al., 2020). After generating ten datasets, one of the ten PVs for reading comprehension provided in the PISA dataset was added to each of these ten datasets. Multicollinearity among the study variables was checked on the basis of the first imputed dataset. The variance inflation factor (VIF) varied from 1.04 to 1.94 across the models and predictors. As VIFs below 2.5 are considered inconspicuous (Johnston et al., 2018) and large standard errors resulting from multicollinearity are particularly problematic in small sample sizes (e.g., Hair et al., 2010), it is unlikely that the results are impaired by multicollinearity.

To ensure meaningful comparisons across groups, measurement invariance needs to be established. Using nonimputed data, which included single items needed for modeling multigroup confirmatory factor analyses, this study investigated measurement invariance across the three groups (minoritized students from highly stigmatized groups, students from other ethnically minoritized groups, majority students) for all central analysis variables. The only exception was reading comprehension, for which only scaled ability estimates (PVs) but no single items or item parcels were available in the dataset. Given the extensive process of item development, validation, and calibration in the PISA, including investigations of measurement bias across countries (OECD, 2020), it can, however, be assumed that the reading comprehension assessment shows measurement equivalence across groups. Scalar measurement invariance could be established for discriminatory climate and a three-dimensional model of teaching quality, comprising classroom management, individual student support, and cognitive activation (see Tables S3–6 in the Supplementary Material). For reading motivation and sense of school belonging, however, the model fit of the least restrictive model probing configural measurement invariance was unacceptable, and the goodness of fit decreased even further for the more restrictive models evaluating metric and scalar invariance (for similar findings for sense of school belonging based on a cross-cultural comparison, see He et al., 2019). Thus, this study refrained from comparing means across groups and did not investigate group-specific relationships between discriminatory climate and these two outcome variables.

To test Hypothesis 1 (differences in perceptions of discriminatory climate on the basis of students’ membership in an ethnically minoritized group), student-reported discriminatory climate was regressed on students’ ethnic backgrounds. Hypothesis 2 (the relationship between perceived discriminatory climate and students’ school adjustment) was tested by running a multilevel regression model with reading comprehension, intrinsic reading motivation, and school belonging as outcome variables. Entering the interaction term between minoritized student status and perceived discriminatory climate allowed the investigation of differential relations between discriminatory climate and school adjustment (i.e., reading comprehension) for the three ethnic groups. Metric Level 1 predictors were grand-mean centered prior to the analyses. In contrast to group-mean centering, this approach allows the estimation of the effects of Level 2 predictors while effectively controlling for variables at Level 1 (Enders & Tofighi, 2007).^6^ The classroom-aggregated and z-standardized variables of discriminatory climate and SES as well as school type were entered as predictors at Level 2. To test Hypothesis 3, the multilevel regression model from Hypothesis 2 was expanded by adding the three dimensions of teaching quality (i.e., classroom management, individual student support, and cognitive activation) as mediators. Classroom management, individual student support, and cognitive activation were entered as mediating variables both at Level 1 and as classroom-aggregated and z-standardized variables at Level 2. To test Hypothesis 4, the multilevel regression model from Hypothesis 2 served as a baseline again. It additionally included the cross-level interaction between the proportion of ethnically minoritized students in the classroom (Level 2) and the discriminatory climate (Level 1), thus exploring whether the relationship between discriminatory climate and school adjustment is moderated by the proportion of minoritized students per classroom^7^.

All analyses were conducted in Mplus 8.10 (Muthén & Muthén, 1998–2023). They included the sampling weight, which corrected for students’ different probabilities of being sampled in the PISA. To consider the multilevel structure of the data, the analyses either used the option “type = complex”, resulting in corrected standard errors (Hypothesis 1), or the option “type = two level”, when predictors at Level 2 were included in the models (Hypotheses 2–4). The mediating role of the three dimensions of teaching quality was investigated by entering them as further dependent variables and by using the “model indirect” command, resulting in indirect, direct, and total (i.e., sum of indirect and direct effects) effects. Using the option “type = imputation” delivered pooled results for the ten datasets. Model fit was evaluated on the basis of the root mean square error of approximation (RMSEA), the comparative fit index (CFI), and the standardized root mean square residual (SRMR). RMSEA values ≤ 0.06, CFI values ≥ 0.95, and SRMR values ≤ 0.08 are typically considered indicators of good model fit (Hu & Bentler, 1999). Fit indices were not available if the models were saturated (i.e., for Model 1 investigating Hypothesis 1) or for random effects modeling (i.e., for Models 3a, 3b, and 3c investigating Hypothesis 4). Code files for data preparation in SPSS as well as for all analyses in R and Mplus can be retrieved from OSF via https://osf.io/rs64u/.

#### Deviations from preregistration

Following advice from the anonymous reviewers, some decisions regarding the analytical procedure were adjusted in revising the manuscript and were, thus, not included in the original analytic plan in the preregistration. This concerns (1) an investigation of measurement invariance for all relevant constructs (if possible with the data at hand), (2) the interaction between perceived discriminatory climate and ethnic group membership in predicting school adjustment, and (3) the inclusion of the proportion of minoritized students per classroom as a control variable in all analyses (and not just as a predictor variable for investigating Hypothesis 4).

## Results

### Descriptive Statistics and Bivariate Correlations

The descriptive statistics and bivariate correlations of the study variables at the student and classroom levels are shown in Tables S7 and S8 in the Supplementary Material. Table S9 shows the descriptive statistics separately for both the ethnically minoritized groups and the ethnic majority group. For the overall sample, the mean perceived discriminatory climate score (*M* = 1.74, *SD* = 0.62) was below the theoretical mean of 2.5, indicating that, overall, students reported experiencing low to intermediate levels of discriminatory climate. Discriminatory climate was negatively associated with all three indicators of school adjustment (i.e., reading comprehension, intrinsic reading motivation, and sense of school belonging) and with student-reported classroom management. Older students, male students, students who mostly spoke a language other than German at home, and students with lower SES perceived teachers at their school as more discriminatory than their counterparts did. School track was substantially correlated with all other study variables at Level 2, confirming previous research that highlighted the differential learning environments in academic and nonacademic track schools in the German school system (Trautwein et al., 2015). Specifically, teachers were perceived as less discriminatory at academic track schools than at nonacademic track schools, and while the proportion of ethnically minoritized students was smaller, students from families with a high SES were overrepresented at academic track schools. Compared with students at nonacademic track schools, those at academic track schools reported better classroom management but less individual student support and cognitive activation. While ethnically minoritized students did not differ from ethnic majority students regarding their perceptions of classroom management, both ethnically minoritized students from stigmatized groups and those from other countries reported higher levels of individual student support and cognitive activation than did ethnic majority students (Table S9).

### Differences in Perceptions of Discriminatory Climate by Student Ethnic Background

The results of the multiple linear regression analyses for explaining the student perceived discriminatory climate are shown in Table 1. In line with Hypothesis 1, minoritized students from Turkey, the SWANA region, sub-Saharan Africa, or Kurdish areas, who were assumed to be strongly stigmatized in German society, indeed perceived a stronger discriminatory climate than ethnic majority students and minoritized students from other countries did (for descriptive findings, see Table S9). Moreover, both gender and school track significantly contributed to explaining differences in discriminatory climate, with male students reporting higher levels of discriminatory climate than female students and students at academic track schools perceiving a less discriminatory climate than students at nonacademic track schools.

**Table 1:** Results of regression analysis predicting discriminatory climate

	Model 1				
	ß	*SE*	*95% CI*		*p*
			*LL*	*UL*	
Minoritized student status (Ref.: minoritized students from stigmatized ethnic groups)					
Ethnic majority	−0.12**	0.04	−0.21	−0.04	0.005
Students from other ethnically minoritized groups	−0.09*	0.04	−0.16	−0.02	0.011
Gender	0.07**	0.02	0.02	0.11	0.004
Age	0.02	0.02	−0.03	0.07	0.455
Language spoken at home	0.05	0.03	−0.01	0.11	0.115
SES^a^	−0.01	0.02	−0.06	0.04	0.631
School track	−0.13***	0.03	−0.18	−0.08	<0.001
*R*^2^	0.04**				<0.001

The group of “minoritized students from stigmatized ethnic groups” included minoritized students with heritage from Turkey, the SWANA region, sub-Saharan Africa, and Kurdish areas. The group of “students from other ethnically minoritized groups” comprised minoritized students from any other country. Gender is coded as 0 = girl, 1 = boy. Language spoken at home is coded as 0 = only or mostly German spoken at home, 1 = mostly a language other than German. The school track is coded as 0 = another school track, 1 = academic school track

*ß* standardized regression coefficient, *SE* standard error, *CI* confidence interval

*p < 0.05; **p < 0.01; ***p < 0.001

^a^*SES* Socioeconomic status

### Relationships between Discriminatory Climate and Students’ School Adjustment

In the next step, this study investigated the role of a discriminatory climate in explaining students’ school adjustment via multilevel regression analyses. Owing to the lack of measurement invariance for reading motivation and sense of school belonging, the variables of minoritized student status as well as their interaction terms with discriminatory climate were only entered as predictors in the model explaining reading comprehension. Discriminatory climate emerged as a significant negative predictor of reading comprehension (Model 1a in Table 2), reading motivation (Model 1b in Table 4), and sense of school belonging (Model 1c in Table 5), thus confirming Hypothesis 2. However, as indicated by the nonsignificant interaction terms in Model 1a (Interact 1) and Model 1a (Interact 2) in Table 2, the effect of perceived discriminatory climate on reading comprehension did not differ across ethnic groups. While the main effect of discriminatory climate as assessed at the student level (Level 1) was found for all three dependent variables, additional effects of the classroom-level aggregates were observed only for students’ reading comprehension (Model 1a in Table 2). Thus, students from classrooms who perceived a more discriminatory climate performed more poorly on the reading comprehension test than did students from classrooms who perceived a less discriminatory climate, even when considering students’ individual perceptions of discriminatory climate along with important control variables at the student and classroom levels (e.g., gender, SES, school track). Students from the most stigmatized student group performed more poorly on the reading comprehension test than did their ethnic majority counterparts. The model fit was very good (see Model 1a, Model 1a [Interact 1], Model 1a [Interact 2], Model 1b, and Model 1c). Whereas the amount of explained variance at Level 1 was comparably high for reading comprehension (*R*^2^ = 0.17) and reading motivation (*R*^2^ = 0.13), it was quite low for sense of belonging to school (*R*^2^ = 0.01).^8^

**Table 2:** Results of regression analyses predicting reading comprehension, considering minoritized student status

	Model 1a (Baseline)					Model 1a (Interact 1)					Model 1a (Interact 2)				
	ß	*SE*	*95% CI*		*p*	ß	*SE*	*95% CI*		*p*	ß	*SE*	*95% CI*		*p*
			*LL*	*UL*				*LL*	*UL*				*LL*	*UL*	
*Student level (L1)*															
Minoritized student status (Ref.: minoritized students from stigmatized ethnic groups)															
Ethnic majority	0.14***	0.03	0.07	0.20	<0.001	0.14***	0.04	0.07	0.20	<0.001	0.14***	0.03	0.07	0.21	<0.001
Students from other ethnically minoritized groups	0.07*	0.03	0.01	0.13	0.030	0.06*	0.03	0.00	0.12	0.037	0.07*	0.03	0.01	0.13	0.023
Ethnic majority x discriminatory climate						0.03	0.09	−0.14	0.21	0.705					
Other ethnically minoritized groups x discriminatory climate											−0.06	0.07	−0.19	0.07	0.373
Gender	−0.10***	0.02	−0.14	−0.07	<0.001	−0.10***	0.02	−0.14	−0.07	<0.001	−0.10***	0.02	−0.14	−0.07	<0.001
Age	−0.14***	0.02	−0.18	−0.09	<0.001	−0.14***	0.02	−0.18	−0.09	<0.001	−0.14***	0.02	−0.18	−0.09	<0.001
Language spoken at home	−0.13***	0.03	−0.18	−0.08	<0.001	−0.13***	0.03	−0.19	−0.09	<0.001	−0.13***	0.03	−0.18	−0.08	<0.001
SES^a^	0.07*	0.03	0.00	0.13	0.047	0.07*	0.03	0.00	0.13	0.046	0.07*	0.03	0.00	0.13	0.045
Discriminatory climate	−0.21***	0.02	−0.26	−0.17	<0.001	−0.25**	0.09	−0.43	−0.06	0.008	−0.16*	0.07	−0.28	−0.03	0.018
*Classroom level (L2)*															
School track	0.50***	0.09	0.32	0.67	<0.001	0.50***	0.09	0.33	0.67	<0.001	0.50***	0.09	0.33	0.67	<0.001
Discriminatory climate (M)	−0.14*	0.06	−0.26	−0.02	0.028	−0.14*	0.06	−0.26	−0.02	0.028	−0.14*	0.06	−0.26	−0.02	0.029
SES (M)	0.42**	0.14	0.15	0.69	0.002	0.42**	0.14	0.15	0.69	0.002	0.42**	0.14	0.15	0.69	0.002
Proportion of minoritized students	0.04	0.09	−0.13	0.21	0.655	0.04	0.09	−0.13	0.21	0.653	0.04	0.09	−0.13	0.21	0.647
*R*^2^ (Level 1)	0.17***				<0.001	0.17***				<0.001	0.17***				<0.001
*R*^2^ (Level 2)	0.78***				<0.001	0.78***				<0.001	0.78***				<0.001
Ϫ*R*^2^															
*ICC*	0.35					0.35					0.35				
*Model fit information*															
RMSEA	0.03					0.03					0.03				
CFI	0.99					0.99					0.99				
SRMR_within_	0.01					0.01					0.01				
SRMR_between_	0.02					0.02					0.03				

The group of “minoritized students from stigmatized ethnic groups” included minoritized students with heritage from Turkey, the SWANA region, sub-Saharan Africa, and Kurdish areas. The group of “students from other ethnically minoritized groups” comprised minoritized students from any other country. Gender is coded as 0 = girl, 1 = boy. Language spoken at home is coded as 0 = only or mostly German spoken at home, 1 = mostly a language other than German. The school track is coded as 0 = another school track, 1 = academic school track

*ß* standardized regression coefficient, *SE* standard error, *CI* confidence interval

*p < 0.05; **p < 0.01; ***p < 0.001.

^a^*SES* Socioeconomic status. *ICC* intraclass correlation. The proportion of minoritized students refers to all ethnically minoritized students in class (i.e., minoritized students from stigmatized ethnic groups and students from other minoritized ethnic groups)

### The Mediating Role of Teaching Quality

Subsequent analyses targeting the main goal of the study investigated whether the negative relationship between a discriminatory climate and students’ school adjustment is mediated by teaching quality, including classroom management, individual student support, and cognitive activation as mediators at the student and classroom levels (Model 2a in Table 3, Model 2b in Table 4, and Model 2c in Table 5). All three dimensions of teaching quality, as measured at the student level, emerged as significant predictors of students’ school adjustment. While individual student support was positively associated with all three outcome variables, classroom management was positively related to students’ reading comprehension and sense of school belonging. In contrast to expectations, however, cognitive activation was negatively related to students’ reading comprehension, indicating that students whose teachers formulated clear learning goals and closely monitored their students’ understanding and learning progress performed more poorly on the reading comprehension test than did students in German classrooms with less cognitive activation. Additionally, the classroom-level aggregate of classroom management was significantly (and positively) associated with students’ reading comprehension.

**Table 3:** Results of regression analyses predicting reading comprehension

	Model 2a					Model 3a^b^				
	ß	*SE*	*95% CI*		*p*	*B*	*SE*	*95% CI*		*p*
			*LL*	*UL*				*LL*	*UL*	
*Student level (L1)*										
Minoritized student status (Ref.: minoritized students from stigmatized ethnic groups)										
Ethnic majority	0.13***	0.03	0.07	0.20	<0.001	29.80***	7.10	15.88	43.73	<0.001
Students from other ethnically minoritized groups	0.06*	0.03	0.01	0.12	0.032	17.84**	6.83	4.45	31.23	0.009
Gender	−0.09***	0.02	−0.13	−0.06	<0.001	−14.63***	2.78	−20.10	−9.17	<0.001
Age	−0.14***	0.02	−0.18	−0.09	<0.001	−15.73***	2.71	−21.03	−10.43	<0.001
Language spoken at home	−0.13***	0.03	−0.18	−0.08	<0.001	−23.75***	5.41	−34.35	−13.15	<0.001
SES^a^	0.07*	0.03	0.00	0.13	0.039	4.48	2.58	−0.57	9.53	0.082
Discriminatory climate	−0.20***	0.02	−0.24	−0.16	<0.001	−16.83***	1.81	−20.37	−13.29	<0.001
Classroom management	0.07**	0.02	0.03	0.12	0.003					
Individual student support	0.05*	0.02	0.01	0.10	0.020					
Cognitive activation	−0.07**	0.02	−0.12	−0.02	0.004					
*Classroom level (L2)*										
School track	0.46***	0.09	0.29	0.63	<0.001	55.66***	11.21	33.69	77.62	<0.001
Discriminatory climate *(M)*	−0.13*	0.07	−0.26	0.00	0.047	−7.42*	3.30	−13.88	−0.96	0.024
SES *(M)*	0.41**	0.13	0.16	0.67	0.002	21.30**	7.08	7.41	35.18	0.003
Classroom management	0.13*	0.06	0.01	0.25	0.030					
Individual student support	−0.12	0.07	−0.25	0.02	0.094					
Cognitive activation	−0.12	0.08	−0.26	0.03	0.116					
Proportion of minoritized students	0.07	0.09	−0.10	0.24	0.415	2.21	4.33	−6.27	10.70	0.609
*Cross-level interaction*										
Discriminatory climate (Level 1) x proportion of minoritized students (Level 2)						−0.08	1.93	−3.71	3.87	0.968
*R*^2^ (Level 1)	0.18***				<0.001					
*R*^2^ (Level 2)	0.79***				<0.001					
Ϫ*R*^*2*^	0.01 (comp. with Model 1a)									
*ICC*	0.34						0.35			
*Model fit information*										
RMSEA	0.11									
CFI	0.56									
SRMR_within_	0.08									
SRMR_between_	0.16									

The group of “minoritized students from stigmatized ethnic groups” included minoritized students with heritage from Turkey, the SWANA region, sub-Saharan Africa, and Kurdish areas. The group of “students from other ethnically minoritized groups” comprised minoritized students from any other country. Gender is coded as 0 = girl, 1 = boy. Language spoken at home is coded as 0 = only or mostly German spoken at home, 1 = mostly a language other than German. The school track is coded as 0 = another school track, 1 = academic school track

*ß* standardized regression coefficient, *SE* standard error, *CI* confidence interval, *B* unstandardized regression coefficient

*p < 0.05; **p < 0.01; ***p < 0.001

^a^*SES* Socioeconomic status. *ICC* intraclass correlation. The proportion of minoritized students refers to all ethnically minoritized students in class (i.e., minoritized students from stigmatized ethnic groups and students from other minoritized ethnic groups)

^b^Standardized regression coefficients, *R*^2^, and model fit indices are not reported by Mplus when conducting cross-level interactions using sample weights and multiply imputed data. Thus, only information on unstandardized regression coefficients is displayed for the respective model

**Table 4:** Results of regression analyses predicting reading motivation

	Model 1b					Model 2b					Model 3b^b^				
	ß	*SE*	*95% CI*		*p*	ß	*SE*	*95% CI*		*p*	*B*	*SE*	*95% CI*		*p*
			*LL*	*UL*				*LL*	*UL*				*LL*	*UL*	
*Student level (L1)*															
Gender	−0.30***	0.02	−0.33	−0.26	<0.001	−0.30***	0.02	−0.33	−0.26	<0.001	−0.69***	0.05	−0.78	−0.60	<0.001
Age	−0.03	0.02	−0.06	0.01	0.183	−0.03	0.02	−0.07	0.01	0.103	−0.05	0.04	−0.12	0.02	0.169
Language spoken at home	0.03	0.02	−0.01	0.07	0.172	0.03	0.02	−0.01	0.07	0.162	0.08	0.07	−0.05	0.21	0.214
SES^a^	0.14**	0.04	0.05	0.22	0.001	0.14**	0.04	0.05	0.22	0.001	0.15*	0.06	0.03	0.26	0.013
Discriminatory climate	−0.08**	0.02	−0.13	−0.04	0.001	−0.08**	0.02	−0.12	−0.03	0.002	−0.11***	0.03	−0.16	−0.05	<0.001
Classroom management						0.01	0.02	−0.03	0.05	0.607					
Individual student support						0.10***	0.02	0.05	0.14	<0.001					
Cognitive activation						0.00	0.02	−0.04	0.05	0.922					
*Classroom level (L2)*															
School track	0.44**	0.13	0.18	0.70	0.001	0.41**	0.13	0.15	0.67	0.002	0.27**	0.09	0.10	0.45	0.002
Discriminatory climate *(M)*	−0.08	0.15	−0.36	0.21	0.592	−0.02	0.13	−0.28	0.24	0.872	−0.02	0.04	−0.11	0.06	0.586
SES *(M)*	0.24	0.16	−0.06	0.55	0.121	0.24	0.15	−0.05	0.53	0.106	0.07	0.05	−0.02	0.16	0.144
Classroom management						0.19	0.12	−0.04	0.43	0.111					
Individual student support						−0.24	0.13	−0.49	0.02	0.065					
Cognitive activation						0.06	0.14	−0.21	0.33	0.654					
Proportion of minoritized students	0.29*	0.13	0.03	0.55	0.029	0.28*	0.13	0.03	0.53	0.031	0.08*	0.04	0.00	0.15	0.038
*Cross-level interaction*															
Discriminatory climate (Level 1) x proportion of minoritized students (Level 2)											0.05	0.03	−0.01	0.10	0.089
*R*^2^ (Level 1)	0.13***				<0.001	0.14***				<0.001					
*R*^2^ (Level 2)	0.39***				<0.001	0.44***				<0.001					
Ϫ*R*^*2*^						0.01 (comp. with Model 1b)									
*ICC*	0.06					0.06									
*Model fit information*															
RMSEA	0.03					0.11									
CFI	0.99					0.56									
SRMR_within_	0.01					0.08									
SRMR_between_	0.02					0.16									

The group of “minoritized students from stigmatized ethnic groups” included minoritized students with heritage from Turkey, the SWANA region, sub-Saharan Africa, and Kurdish areas. The group of “students from other ethnically minoritized groups” comprised minoritized students from any other country. Gender is coded as 0 = girl, 1 = boy. Language spoken at home is coded as 0 = only or mostly German spoken at home, 1 = mostly a language other than German. The school track is coded as 0 = another school track, 1 = academic school track

*ß* standardized regression coefficient, *SE* standard error, *CI* confidence interval, *B* unstandardized regression coefficient

*p < 0.05; **p < 0.01; ***p < 0.001

^a^*SES* Socioeconomic status. *ICC* intraclass correlation. The proportion of minoritized students refers to all ethnically minoritized students in class (i.e., minoritized students from stigmatized ethnic groups and students from other minoritized ethnic groups)

^b^Standardized regression coefficients, *R*^2^, and model fit indices are not reported by Mplus when conducting cross-level interactions using sample weights and multiply imputed data. Thus, only information on unstandardized regression coefficients is displayed for the respective model

**Table 5:** Results of regression analyses predicting school belonging

	Model 1c					Model 2c					Model 3c^b^				
	ß	*SE*	*95% CI*		*p*	ß	*SE*	*95% CI*		*p*	*B*	*SE*	*95% CI*		*p*
			*LL*	*UL*				*LL*	*UL*				*LL*	*UL*	
*Student level (L1)*															
Gender	0.05*	0.02	0.01	0.10	0.028	0.06**	0.02	0.02	0.11	0.008	0.09	0.05	0.00	0.19	0.050
Age	−0.04	0.03	−0.09	0.01	0.140	−0.04	0.02	−0.09	0.00	0.072	−0.06	0.04	−0.13	0.02	0.162
Language spoken at home	−0.00	0.02	−0.05	0.04	0.943	0.00	0.02	−0.05	0.05	0.992	−0.02	0.06	−0.14	0.10	0.725
SES^a^	0.04	0.03	−0.02	0.10	0.150	0.05	0.03	−0.01	0.10	0.128	0.04	0.03	−0.02	0.10	0.186
Discriminatory climate	−0.08**	0.02	−0.13	−0.03	0.002	−0.06*	0.03	−0.11	−0.01	0.011	−0.03**	0.03	−0.16	−0.03	0.002
Classroom management						0.12***	0.03	0.06	0.17	<0.001					
Individual student support						0.07*	0.03	0.01	0.12	0.016					
Cognitive activation						0.02	0.03	−0.03	0.07	0.474					
*Classroom level (L2)*															
School track	0.22	0.21	−0.18	0.62	0.285	0.13	0.24	−0.33	0.59	0.579	0.06	0.06	−0.06	0.19	0.302
Discriminatory climate *(M)*	−0.23	0.26	−0.74	0.27	0.362	−0.19	0.27	−0.72	0.34	0.484	−0.03	0.03	−0.10	0.03	0.318
SES *(M)*	0.25	0.27	−0.06	0.55	0.359	0.25	0.30	−0.34	0.85	0.403	0.03	0.04	−0.04	0.10	0.386
Classroom management						0.09	0.26	−0.43	0.60	0.739					
Individual student support						−0.13	0.29	−0.70	0.45	0.672					
Cognitive activation						−0.27	0.32	−0.91	0.36	0.397					
Proportion of minoritized students	0.22	0.21	−0.19	0.62	0.303	0.23	0.25	−0.25	0.71	0.340	0.03	0.03	−0.02	0.08	0.269
*Cross*−*level interaction*															
Discriminatory climate (Level 1) x proportion of minoritized students (Level 2)											0.01	0.03	−0.04	0.05	0.824
*R*^2^ (Level 1)	0.01*				0.026	0.04***				<0.001					
*R*^2^ (Level 2)	0.30				0.175	0.38				0.225					
Ϫ*R*^*2*^						0.03 (comp. with Model 1c)									
*ICC*	0.02					0.02									
*Model fit information*															
RMSEA	0.03					0.11									
CFI	0.99					0.56									
SRMR_within_	0.01					0.08									
SRMR_between_	0.02					0.16									

The group of “minoritized students from stigmatized ethnic groups” included minoritized students with heritage from Turkey, the SWANA region, sub-Saharan Africa, and Kurdish areas. The group of “students from other ethnically minoritized groups” comprised minoritized students from any other country. Gender is coded as 0 = girl, 1 = boy. Language spoken at home is coded as 0 = only or mostly German spoken at home, 1 = mostly a language other than German. The school track is coded as 0 = another school track, 1 = academic school track

*ß* standardized regression coefficient, *SE* standard error, *CI* confidence interval, *B* unstandardized regression coefficient

*p < 0.05; **p < 0.01; ***p < 0.001

^a^*SES* Socioeconomic status. *ICC* intraclass correlation. The proportion of minoritized students refers to all ethnically minoritized students in class (i.e., minoritized students from stigmatized ethnic groups and students from other minoritized ethnic groups)

^b^Standardized regression coefficients, *R*^2^, and model fit indices are not reported by Mplus when conducting cross-level interactions using sample weights and multiply imputed data. Thus, only information on unstandardized regression coefficients is displayed for the respective model

The paths from the discriminatory climate to teaching quality as well as all total, indirect, and direct paths from the mediation models are displayed in Table 6 (for reading comprehension), Table 7 (for reading motivation), and Table 8 (for sense of school belonging). There was a significant relationship between discriminatory climate and classroom management (β = −0.16, *p* < 0.001, *SE* = 0.02; Level 1), suggesting that students who perceived a more discriminatory climate perceived the classroom management as less favorable (i.e., more disturbances, less efficient use of time) than their counterparts did. Additionally, there was a trend toward lower levels of perceived individual student support (β = −0.04, *p* = 0.052, *SE* = 0.02; Level 1). Investigating indirect effects further revealed that the negative relationships of a discriminatory climate with reading comprehension and sense of school belonging were indeed (partially) mediated by classroom management (see indirect paths at Level 1 in Tables 6 and 8) with the direct effects remaining statistically significant. None of the other dimensions of teaching quality acted as mediators between discriminatory climate and students’ school adjustment, thus only partially supporting the hypothesis. The suggested mediation model, thus, did not fit the data very well as reflected in the poor model fit indices (Model 2a in Table 3, Model 2b in Table 4, and Model 2c in Table 5). The increase in the amount of explained variance was small and ranged from Ϫ*R*^2^ = 0.01 for reading comprehension and reading motivation to Ϫ*R*^2^ = 0.03 for sense of school belonging.

**Table 6:** Results of the mediation analysis of discriminatory climate via student-reported teaching quality on students’ reading comprehension (N = 2947)

Effect	Path	Model 2a Mediating variable: Classroom management					Model 2a Mediating variable: Individual student support					Model 2a Mediating variable: Cognitive activation				
		β	*SE*	*95% CI*		*p*	β	*SE*	*95% CI*		*p*	β	*SE*	*95% CI*		*p*
				*LL*	*UL*				*LL*	*UL*				*LL*	*UL*	
	*Student level (L1)*															
	Discriminatory climate	−0.16***	0.02	−0.21	−0.11	<0.001	−0.04	0.02	−0.08	0.00	0.052	0.01	0.03	−0.04	0.07	0.693
total	Discriminatory climate → Reading comprehension	−0.22***	0.02	−0.26	−0.17	<0.001										
indirect	Discriminatory climate → Teaching quality → Reading comprehension	−0.01**	0.00	−0.02	0.00	0.007	0.00	0.00	−0.01	0.00	0.143	0.00	0.00	−0.01	0.00	0.711
direct	Discriminatory climate → Reading comprehension	−0.20***	0.02	−0.24	0.16	<0.001										
	*Classroom level (L2)*															
	Discriminatory climate	−0.25	0.21	−0.67	0.18	0.257	0.12	0.23	−0.33	0.58	0.599	0.06	0.08	−0.09	0.21	0.418
total	Discriminatory climate *(M)* → Reading comprehension	−0.19**	0.07	−0.31	−0.06	0.004										
indirect	Discriminatory climate *(M)* → Teaching quality *(M)* → Reading comprehension	−0.03	0.03	−0.10	0.03	0.298	−0.02	0.03	−0.09	0.06	0.669	−0.01	0.01	−0.03	0.01	0.493
direct	Discriminatory climate *(M)* → Reading comprehension	−0.13*	0.07	−0.26	0.00	0.047										

*p < 0.05; **p < 0.01; ***p < 0.001

**Table 7:** Results of the mediation analysis of discriminatory climate via student-reported teaching quality on students’ reading motivation (N = 2947)

Effect	Path	Model 2b Mediating variable: Classroom management					Model 2b Mediating variable: Individual student support					Model 2b Mediating variable: Cognitive activation				
		β	*SE*	*95% CI*		*p*	β	*SE*	*95% CI*		*p*	β	*SE*	*95% CI*		*p*
				*LL*	*UL*				*LL*	*UL*				*LL*	*UL*	
	*Student level (L1)*															
	Discriminatory climate	−0.16***	0.02	−0.21	−0.11	<0.001	−0.04	0.02	−0.08	0.00	0.052	0.01	0.03	−0.04	0.07	0.693
total	Discriminatory climate → Reading motivation	−0.08**	0.02	−0.13	−0.03	0.001										
indirect	Discriminatory climate → Teaching quality → Reading motivation	0.00	0.00	−0.01	0.01	0.610	0.00	0.00	−0.01	0.00	0.073	0.00	0.00	0.00	0.00	0.928
direct	Discriminatory climate → Reading motivation	−0.08**	0.02	−0.12	−0.03	0.002										
	*Classroom level (L2)*															
	Discriminatory climate	−0.25	0.21	−0.67	0.18	0.257	0.12	0.23	−0.33	0.58	0.599	0.06	0.08	−0.09	0.21	0.418
total	Discriminatory climate *(M)* → Reading motivation	−0.10	0.13	−0.34	0.15	0.447										
indirect	Discriminatory climate *(M)* → Teaching quality *(M)* → Reading motivation	−0.05	0.06	−0.16	0.06	0.390	−0.03	0.07	−0.16	0.10	0.721	0.00	0.01	−0.02	0.03	0.731
direct	Discriminatory climate *(M)* → Reading motivation	−0.02	0.13	−0.28	0.24	0.872										

*p < 0.05; **p < 0.01; ***p < 0.001

**Table 8:** Results of the mediation analysis of discriminatory climate via student-reported teaching quality on students’ sense of belonging (N = 2947)

Effect	Path	Model 2c Mediating variable: Classroom management					Model 2c Mediating variable: Individual student support					Model 2c Mediating variable: Cognitive activation				
		β	*SE*	*95% CI*			β	*SE*	*95% CI*			β	*SE*	*95% CI*		
				*LL*	*UL*	*p*			*LL*	*UL*	*p*			*LL*	*UL*	*p*
	*Student level (L1)*															
	Discriminatory climate	−0.16***	0.02	−0.21	−0.11	<0.001	−0.04	0.02	−0.08	0.00	0.052	0.01	0.03	−0.04	0.07	0.693
total	Discriminatory climate → Sense of belonging	−0.08**	0.03	−0.13	−0.04	0.001										
indirect	Discriminatory climate → Teaching quality → Sense of belonging	−0.02**	0.00	−0.03	−0.01	<0.001	0.00	0.00	−0.01	0.00	0.141	0.00	0.00	0.00	0.00	0.822
direct	Discriminatory climate → Sense of belonging	−0.06**	0.03	−0.11	−0.01	0.011										
	*Classroom level (L2)*															
	Discriminatory climate	−0.25	0.21	−0.67	0.18	0.257	0.12	0.23	−0.33	0.58	0.599	0.06	0.08	−0.09	0.21	0.418
total	Discriminatory climate *(M)* → Sense of belonging	−0.25	0.26	−0.76	0.26	0.339										
indirect	Discriminatory climate *(M)* → Teaching quality *(M)* → Sense of belonging	−0.03	0.09	−0.20	0.14	0.754	−0.02	0.03	−0.14	0.11	0.609	−0.19	0.27	−0.08	0.05	0.484
direct	Discriminatory climate *(M)* → Sense of belonging	−0.19	0.27	−0.72	0.34	0.283										

*p < 0.05; **p < 0.01; ***p < 0.001

### The Buffering Effect of the Proportion of Ethnically Minoritized Students

Finally, this study explored the role of the proportion of ethnically minoritized students as a potential buffer in the relationship between discriminatory climate and students’ school adjustment. Building on the baseline multilevel models (Model 1a in Table 2, Model 1b in Table 4, Model 1c in Table 5), which included the proportion of students from minoritized ethnic groups as a control variable, a cross-level interaction of perceived discriminatory climate at Level 1 and the proportion of minoritized students at Level 2 were therefore added as further predictors of school adjustment (Model 3a in Table 3, Model 3b in Table 4, Model 3c in Table 5). Whereas the proportion of ethnically minoritized students in class was unrelated to students’ reading comprehension (Model 3a) and sense of school belonging (Model 3c), a significant and positive effect emerged for reading motivation (Model 3b), suggesting that a greater share of ethnically minoritized students in class was associated with increased reading motivation. No statistically significant cross-level interaction emerged for any of the outcome variables, indicating that the effect of perceived discriminatory climate did not vary depending on the share of ethnically minoritized students in class. Thus, contrary to the assumption, the negative relationship between a discriminatory climate and the variables of students’ school adjustment was not weakened in the presence of a larger proportion of ethnically minoritized students in the classroom.

## Discussion

As classrooms in many countries, including Germany, are marked by an increasingly ethnically diverse student body, it is important to gain a clear understanding of what helps and hinders the school adaptation of ethnically minoritized students in these contexts. Previous research has highlighted the detrimental effects of personal ethnic-racial discrimination on minoritized students’ well-being and academic performance (Civitillo et al., 2023) and initial evidence points to negative relationships between a discriminatory climate and the adjustment of both ethnically minoritized and majority students (Baysu et al., 2023). However, less is known about the classroom-level processes and mechanisms that contribute to this relationship (Denessen et al., 2022). Using nationally representative data from secondary school students in Germany, this study investigated the relationship between a discriminatory climate and adolescents’ school adjustment, thus corroborating prior research (Baysu et al., 2023). This study adds to previous findings by considering (1) teachers’ classroom behavior as reflected in the basic dimensions of teaching quality as a mediating variable and (2) classroom ethnic composition as a moderating variable in the relation between a discriminatory climate and adolescents’ academic adjustment. Moreover, using data from Germany allowed for investigating differences in student-perceived discriminatory climate across two groups of ethnically minoritized students as well as differential relationships between discriminatory climate and academic outcomes. Prior research on personal discrimination heavily relied on data from U.S.-based samples and respective minoritized groups (i.e., Black students; Civitillo et al., 2023), whereas initial findings on discriminatory climate used international student data that do not allow for the identification subgroups of ethnically minoritized students (Baysu et al., 2023). This study, thus, contributes to a more nuanced picture of a discriminatory climate and its relationships with academic adjustment.

### Higher Perceived Discriminatory Climate among Minoritized Students from Highly Stigmatized Groups

In line with Hypothesis 1 and previous research focusing on adolescent samples in Germany (Schotte et al., 2024; Vietze et al., 2023), ninth graders from presumably strongly stigmatized groups in German society, that is, those from Turkey, the SWANA region, sub-Saharan Africa, or Kurdish areas, not only experienced a more discriminatory school climate than their counterparts from ethnic majority groups did but also experienced a more discriminatory climate than ethnically minoritized students from other countries did (Model 1 in Table 1). As the abovementioned countries of origin largely overlap with countries with predominantly Muslim populations, the students’ higher levels of a perceived discriminatory climate may reflect anti-Muslim attitudes, which are highly salient in public discourse in Germany (e.g., Kaddor et al., 2018). Moreover, minoritized students from stigmatized groups are fairly likely to be visible minorities (e.g., Black Germans with heritage from sub-Saharan Africa). The finding that these students perceive the school climate as particularly discriminatory thus resonates with prior research, suggesting that the risk of experiencing racism and discrimination differs by skin color (DeZIM, 2023). However, importantly, the PISA measure of discriminatory climate does not assess personal discriminatory experiences nor does it focus on specific ethnically minoritized groups (e.g., those from Turkey or the SWANA region); rather, it more generally asks about teachers’ discriminatory attitudes and utterances toward certain ethnic groups. Therefore, with the data at hand, it is not possible to disentangle whether ethnically minoritized students and minoritized youth from stigmatized ethnic groups in particular are generally more susceptible to ethnic-racial discrimination by teachers or whether their different degrees of perceived discrimination actually reflect differences in personal or group-based discrimination.

### Negative Relationships between Discriminatory Climate and Adolescents’ School Adjustment

The results of the multilevel regression analyses confirmed negative relationships between a discriminatory climate, created by teachers’ biased attitudes and overt devaluation of some ethnic groups, and all three measures of adolescents’ school adjustment, hence supporting Hypothesis 2. Specifically, experiences of a discriminatory climate are associated with poorer reading achievement (Model 1a, Model 1a [Interact 1], and Model 1a [Interact 2] in Table 2), less reading motivation (Model 1b in Table 4), and a reduced sense of school belonging in the overall sample (Model 1c in Table 5). This is a compelling finding, considering that it holds when accounting for important confounding variables at the student and classroom levels, such as gender, SES, and school track. These results coincide with a large body of previous work that documented negative links between personal ethnic-racial discrimination by teachers and both academic achievement and socioemotional outcomes (i.e., different indicators of well-being, including sense of school belonging; Civitillo et al., 2023). Moreover, they are in line with initial evidence highlighting that not only personal experiences of discrimination but also instances of witnessing ethnic-racial discrimination, as reflected, for instance, in a discriminatory climate, are accompanied by poor academic achievement (Del Toro et al., 2024). However, previous studies, including those that were considered in recent meta-analytic work (Civitillo et al., 2023), focused on U.S.-based samples and investigated effects on ethnically minoritized students only. An exception is a study which was also based on PISA 2018 data (Baysu et al., 2023). By incorporating data from 60 countries, the authors revealed that both students from ethnically minoritized groups and majority students yielded poorer academic achievement when teachers at their schools were perceived as more discriminatory. In contrast to that study, the present study’s focus on adolescents in Germany allowed to distinguish between different groups of ethnically minoritized students (see Hypothesis 1). In terms of group-specific differences in school adjustment, the analyses revealed poorer reading comprehension among minoritized youth from highly stigmatized ethnic groups than among ethnic majority students and other minoritized students, even when considering a range of variables that contribute to explaining achievement gaps (e.g., differences in SES or attendance of academic vs. nonacademic track schools). While this student group also reported particularly high levels of perceived discriminatory climate (see Hypothesis 1), the relationship between discriminatory climate and reading comprehension did not differ from that of the other two groups. This corresponds to previous findings on the relationship between classroom-level perceptions of discriminatory teacher behavior and the academic adjustment of minoritized youth and majority students (Baysu et al., 2023). It also resonates with some of the research investigating differential relationships between personal discriminatory experiences and academic adjustment for different ethnic groups (for divergent findings, however, see Cokley et al., 2017). While a study with ethnically minoritized college students in the U.S. reported that Black students experienced more discriminatory behavior than Asian American and Latinx college students did, this did not have a stronger detrimental effect on their grades and life satisfaction than it did for their peers (Del Toro & Hughes, 2020). Overall, the present findings highlight that discriminatory teacher behavior and utterances not only jeopardize ethnically minoritized students, who are their direct target, but are also associated with lowered school adjustment in majority students.

### Classroom Management as a Mediator of the Relationship between Discriminatory Climate and Adolescents’ Academic Adjustment

For the mediating role of teaching quality in the association between a discriminatory climate and school adjustment, Hypothesis 3 was only partially supported. Specifically, only the perception of poorer classroom management emerged as a significant mediator in the association with two outcome variables. Adolescents who perceived teachers at their schools as being more discriminatory experienced lessons as less structured and more disruptive, which was negatively associated with their reading comprehension (indirect path of Model 2a in Table 6) and school belonging (indirect path of Model 2c in Table 8). In these classrooms, learning time, thus, seems to be used less efficiently, and students may not feel that they have very favorable learning conditions. This might result from a complex interplay of students’ and teachers’ perceptions and behaviors in the classroom. On the one hand, students who experience their teachers as being biased toward and misinformed about certain ethnic groups might strive against discrimination by opposing their teachers, thus ignoring their instructions and interrupting the lessons. On the other hand, teachers who make derogatory comments about and have lower expectations for ethnically minoritized students are probably less convinced about the potential benefits of well-organized and clearly structured lessons, hence putting less effort into their classroom management. This line of reasoning is supported by research on teacher expectancy effects, showing that teachers’ biased expectations of their students can result in differential treatment (e.g., Gentrup et al., 2020; Wang et al., 2018). Nevertheless, the indirect effects of classroom management were very small in size and the direct effects of the discriminatory climate remained significant when classroom management was considered (direct path of Model 2a in Table 6 and of Model 2c in Table 8). Teachers’ less clear structuring of the lessons and less efficient use of study time, thus, seems to be just one among various processes occurring in the classroom and in the school context that help explain how teachers’ discriminatory utterances and attitudes translate into reduced achievement and sense of school belonging.

In interpreting the results on the mediating role of teaching quality, it needs to be considered that the PISA assessment of teaching quality reflects students’ perceptions of average instructional behavior in the classroom and not specific behavior and support strategies directed toward individual students. This is a common approach for assessing teaching quality, both in student questionnaires (e.g., Henschel, Rjosk, et al., 2023) and in classroom observations (Pianta et al., 2008). Focusing on a classroom-level perspective rather than on individual perceptions of teaching behavior may, however, be more appropriate for certain dimensions of teaching quality than for others. Specifically, whereas classroom management refers to organizational lesson characteristics that should be the same for all students in class, the degree to which students feel supported and respected and perceive the lessons as challenging and cognitively activating may differ widely across students. This may have contributed to the finding that classroom management, but not individual student support and cognitive activation, emerged as significant mediators.

Individual student support was positively associated with all outcome variables and, by trend, negatively linked to the discriminatory climate (*p* = 0.052, see, for instance, Model 2a in Table 6 for the mediating variable of individual student support), which was basically in line with the assumptions. Cognitive activation, however, related negatively to reading achievement, hence contradicting the assumptions. There are two plausible explanations for this finding, considering that cognitive activation was greater at nonacademic track schools than at academic track schools (see bivariate correlations in Table S8) and that students from both ethnically minoritized groups reported more cognitive activation than ethnic majority students did (see descriptive statistics in Table S9). First, whereas students should be challenged by the teachers’ questions and ambitious learning goals, thus helping them engage with lesson content, such teaching behavior might result in feelings of overburdening, if students are not able to accomplish these challenging tasks (for a similar line of reasoning, see Rieser & Decristan, 2023). Second, teaching strategies such as regularly checking students’ understanding or defining their learning goals might be more important for less proficient readers than for more proficient readers. Thus, teachers might indeed use such strategies more often when teaching classrooms with many students who still need to develop their reading comprehension.

### No Buffering Effect of the Proportion of Ethnically Minoritized Students

Finally, Hypothesis 4 was rejected, as classroom ethnic composition did not play a role in the relationship between perceived discriminatory climate and adolescents’ school adjustment (Model 3a in Table 3, Model 3b in Table 4, and Model 3c in Table 5). Thus, the results were not in line with recent findings of a meta-analysis (Civitillo et al., 2023), reporting a buffering effect of classroom ethnic composition on the relationship between personal discrimination by teachers and students’ academic outcomes (for a recent longitudinal study of similar associations, see Baysu et al., 2024). Based on the balance of power hypothesis (D’hondt et al., 2021; Juvonen et al., 2018), which assumes that ethnically minoritized students feel less lonely and less victimized in more ethnically diverse classrooms, an attenuating effect of the proportion of ethnically minoritized students on students’ school adjustment had been expected. Moreover, being taught together with a larger number of minoritized students might result in a greater sense of belonging and greater potential support. However, in line with studies reporting positive effects of an ethnically more diverse student body on students’ socioemotional and motivational outcomes (Demanet et al., 2016; Rjosk et al., 2015), the results of the current study showed that a greater share of ethnically minoritized students in class was associated with increased reading motivation. Although group-specific differences in reading motivation could not be investigated in the present study because of the lack of measurement invariance, this finding aligns with the research strand on immigrant optimism, suggesting that members of ethnically minoritized groups have particularly high educational aspirations and are determined to fare well in the education system (e.g., Sikora & Pokropek, 2021). However, no such effects were found for students’ reading comprehension and sense of school belonging.

In interpreting the results regarding classroom ethnic composition in the present study, several aspects need to be considered. First, while students’ reading comprehension showed substantial variance at the classroom level (intraclass correlation [*ICC*] = 0.35, Table 2), the intraclass correlation was very small for reading motivation (*ICC* = 0.06, Table 4) and negligible for sense of school belonging (*ICC* = 0.02, Table 5). This indicates that only very limited amounts of variance could be explained by classroom-level variables such as classroom ethnic composition in the latter two outcomes. Second, SES and, most importantly, school track were strong classroom-level predictors of students’ reading comprehension and reading motivation. Moreover, these variables were confounded with the proportion of minoritized students per classroom, possibly masking (negative) effects of the classroom composition for reading comprehension. Third, with the data at hand, it was only possible to differentiate between (presumably) highly stigmatized students from ethnically minoritized groups and ethnically minoritized students from other countries, exclusively relying on information about the student’s own and their parents’ country of birth. Thus, it remains unclear whether and to what extent minoritized students recognize their coethnic classmates as such, let alone as individuals of the same ethnic origin, which might be necessary preconditions for perceiving other students as potential supporters and bonding with them. Classifying students as members of ethnically minoritized groups solely on the basis of their own and their parents’ country of birth can generally be viewed as simplistic (cf. German expert commission on the framework conditions for integration capability, 2021). While students with one foreign-born parent may still form part of visible minorities and thus face discrimination in German society, grouping them as members of the ethnic majority did not result in reduced differences in perceived discriminatory climate among the three ethnic groups in the present analyses (see Tables S1 and S9 in the Supplementary Material). However, as the ascription of a minoritized status on the basis of one’s own or one’s parents’ country of birth does not necessarily coincide with a person’s self-identification (Nesterko & Glaesmer, 2019), future studies on the relationship between minoritized student status and perceived discrimination, including a discriminatory climate, might benefit from having students deliberately indicate their ethnic group membership.

### Limitations and Future Directions

This study has several limitations. First, owing to the cross-sectional design implemented in the PISA, conclusions concerning the causal relationships among a discriminatory climate, teaching quality, and students’ school adjustment cannot be drawn. Specifically, the decision to perform a mediation analysis on cross-sectional data was informed mainly by theoretical and conceptual arguments based on the underlying model of teacher‒student interactions in the classroom (Denessen et al., 2022). These assumptions sufficiently back the assumption that teacher stereotypes and discrimination affect teaching quality and not the other way around. However, the assumed timely succession of discriminatory teacher attitudes and utterances, teaching quality, and student outcomes cannot be proven with the data at hand. Second, and relatedly, all the information included in the present analyses was gathered from the students, meaning that no other, possibly more objective information source (e.g., classroom observations for assessing teaching quality), could be considered. Therefore, it cannot be completely ruled out that students with relatively poor academic performance have an overall more negative attitude toward school, resulting in a more negative perception of their teachers’ stereotyping and discriminatory behavior. However, the finding that ethnically minoritized students report even higher values for individual student support and cognitive activation (see the descriptive statistics in Table S9) makes this interpretation rather unlikely. As the bulk of studies on the relationship between experiences of personal discrimination and school adjustment rely on cross-sectional data (for an overview, see Civitillo et al., 2023), future research would benefit from longitudinal studies that allow for a more nuanced assessment of teachers’ classroom practices and behaviors through which stereotypes and discriminatory attitudes affect students’ school adjustment. Third, as indicated above, the analyses included a measure of discriminatory climate but not of personal experiences of discrimination. Likewise, assessments of teaching quality were based on students’ overall perceptions of teacher behavior in the classroom and not on reports of teacher behavior directed toward an individual student. Future research would benefit from (additionally) including individual experiences of discrimination as well as individual perceptions of teacher behavior, as these measures might provide further insights into the mechanisms underlying the relationship between discriminatory teacher behavior and adolescents’ academic adjustment.

### Implications for Teacher Training and School Interventions

The findings of the present study have important implications that should be used for informing and developing teacher training programs in Germany and beyond (Kleen & Glock, 2018). Despite increasing efforts to prepare student teachers for teaching in ethnically diverse classrooms, university teacher training programs in Germany still lack systematic and compulsory courses on multicultural education (Civitillo & Juang, 2019). Such learning opportunities are, however, urgently needed to help future teachers reflect upon their ethnic stereotypes and—possibly unconscious—discriminatory behaviors and to adopt culturally responsive teaching approaches. Teachers’ ability to create respectful and encouraging learning environments for all students, irrespective of their ethnic group membership, has been shown to be positively linked with the achievement (Ialuna et al., 2024) and sense of school belonging (Byrd, 2016) of both ethnically minoritized and majority students. As suggested by the present results, they may also help teachers conduct their lessons more undisturbed and with a more efficient use of study time. Educational policy makers and teacher educators, thus, need to make an effort to create more course offerings in this regard. In addition, schools should establish policies to monitor and address discriminatory practices at an institutional level. Mechanisms for reporting and addressing such behaviors could help foster a safer environment for all students.

## Conclusion

The emerging literature has demonstrated that a discriminatory climate can hamper the school adjustment of both ethnically minoritized and ethnic majority youth, but the classroom processes contributing to this relationship have not yet been investigated. Initial research on personal discrimination in Germany further indicates that minoritized students with Turkish or Arabic heritage as well as Black students face more discrimination in German society and at school than ethnically minoritized students from other groups do. However, it remains largely unclear whether minoritized students from strongly stigmatized ethnic groups also perceive the discriminatory climate differently and whether this, in turn, increases their disadvantages in school adaptation. Using nationally representative data of German ninth graders from the PISA, the present study established that ethnically minoritized students differ in the degree to which they perceive their teachers’ behaviors and utterances as discriminatory, with students with heritage from Turkey, the SWANA region, sub-Saharan Africa, and Kurdish areas perceiving the climate as most discriminatory. The study further demonstrated that a discriminatory climate is negatively associated with different indicators of school adjustment in secondary school students. Importantly, this finding holds for reading comprehension of both ethnically minoritized and majority students and the three investigated ethnic groups are affected to a similar degree. This points not only to vulnerability but also to the attention of adolescents when merely witnessing discriminatory behavior in school without being the explicit target. As adolescence is a developmental period during which teacher‒student relationships are particularly influential for student adjustment and during which perceived discrimination can have pronounced detrimental effects, ensuring a fair and responsive learning climate for all students during secondary schooling is key. Considering the dire need for studies that elucidate the teaching practices that might account for the relationship between discriminatory teacher attitudes and behaviors and student outcomes, this study provides initial evidence that a discriminatory climate is associated with lower teaching quality, which is, in turn, associated with poorer school adjustment. Specifically, the findings highlight the mediating role of poor classroom management, as reflected in a less efficient use of study time and more disruptions.

## Supplementary information

DiscriminatoryClimate_Supplementary Material_final

## Footnotes

1 The term SWANA (South‒West Asia and North Africa) is used as an alternative to the term MENA (Middle East and North Africa) as the latter is rooted in a Eurocentric worldview.

2 Information on the exact countries of birth of students and their parents is not available in the PISA 2018 dataset due to data protection regulations. Therefore, it is not possible for us to report the exact number of students per ethnic group. Grouping of students as “minoritized youth from stigmatized ethnic groups” and as “students from other ethnically minoritized groups” was performed by the Research Data Centre of the IQB based on classification guidelines provided by the authors of the present paper.

3 In additional analyses (not included in the preregistration), students with one foreign-born parent were considered as “minoritized students from highly stigmatized groups” or as “members of other ethnically minoritized groups”, depending on their parent’s country of birth (i.e., they were not considered members of the majority group). This grouping procedure yielded a similar result pattern but, overall, group differences decreased. In particular, when controlling for important confounding variables, group differences in perceived discriminatory climate even disappeared, indicating that this procedure masks differences in perceived discrimination that can be observed among first- and second-generation immigrants but not among students with one foreign-born parent (Tables S1 and S2 in the Supplementary Material).

4 Following the same procedure, an additional variable was created reflecting the proportion of minoritized students from stigmatized ethnic groups and all analyses were rerun using this variable. As this did not alter the result pattern, only the results based on the variable “proportion of minoritized students in the classroom“ are presented in the paper.

5 Using these indicators of SES and educational background separately in the analyses did not alter the overall pattern of results regarding the core research questions.

6 As group-mean centering has been suggested as more suitable for examining cross-level interactions, however (Enders & Tofighi, 2007), all analyses were rerun based on group-mean centering. This did not alter the results.

7 These analyses were also performed with the share of minoritized students from stigmatized ethnic groups (i.e., with heritage from Turkey, the SWANA region, sub-Saharan Africa, and Kurdish areas) which did not alter the results shown in Tables 3–5.

8 Modeling random slopes for discriminatory climate and minoritized student status additionally allowed for checking whether the effects of these variables varied across classrooms. However, as none of the variances of the random slopes were statistically significant, it can be assumed that effects did not differ across classrooms. Analyses presented in this paper are, therefore, based on models with fixed slopes.
