# Supplementary material for: Discriminatory Climate and School Adjustment in Ethnically Minoritized Adolescents and Majority Adolescents: An Investigation of the Mediating Role of Teaching Quality
Source: J Youth Adolesc. 2025 Feb 7;54(7):1732–55. doi: 10.1007/s10964-025-02147-2 (PMC12245938; doi:10.1007/s10964-025-02147-2)
Supplement: Supplementary file 1 — DiscriminatoryClimate_Supplementary Material_final [file 10964_2025_2147_MOESM1_ESM.pdf]

Table S1

Descriptive Statistics of Variables at the Student Level (by Student Group, Based on a Different Grouping Procedure for Students with one Foreign-Born Parent)

		Ethnic majority students		Minoritized students from stigmatized ethnic groups		Students from other ethnically minoritized groups	
		N = 1,916		*N* = 306		*N* = 702	
		M	*SD*	*M*	*SD*	*M*	*SD*
1)	Discriminatory climate	1.68 [1,2]	0.59	1.92 [2]	0.64	1.76	0.60
2)	Reading comprehension	514.86 [1,2]	92.59	440.43 [2]	89.44	471.44	102.62
3)	Intrinsic reading motivation [a]	--	--	--	--	--	--
4)	Sense of school belonging [a]	--	--	--	--	--	--
5)	Classroom management	2.92	0.72	2.88	0.69	2.93	0.70
6)	Individual student support	2.85 [1,2]	0.84	3.01	0.78	2.97	0.84
7)	Cognitive activation	2.70 [1,2]	0.68	2.86	0.66	2.85	0.69
8)	Age	15.38 [1,2]	0.55	15.70 [2]	0.79	15.62	0.75
9)	Gender	51.0%	--	52.9%	--	52.4%	--
10)	Language spoken at home	1.3% [1,2]	--	54.6% [2]	--	45.6%	--
11)	SES [b]	0.13 [1,2]	0.90	-0.80 [2]	1.07	-0.29	0.98
12)	School track	42.4% [1,2]	--	25.2%	--	32.5%	--

Note. Different from the analyses in the main body of the manuscript, an alternative grouping procedure for students with one foreign-born parent was used in these analyses. Specifically, these students were considered as either minoritized students from stigmatized ethnic groups or as students from other ethnically minoritized groups based on their parent's country of birth. Analyses are based on the first imputed dataset. N_{L1} = 2,924. [a]As scalar measurement invariance across groups was not given for this measure and means can, thus, not reasonably be compared across groups, no group-specific means are displayed here. [b]SES = Socioeconomic status. Gender is coded as 0 = girl and 1 = boy. Language spoken at home is coded as 0 = only or mostly German and 1 = mostly a language other than German. The school track is coded as 0 = nonacademic track and 1 = academic track.

[1] = coefficient differs significantly from the one observed for minoritized students from stigmatized ethnic groups (i.e., with heritage from Turkey, the SWANA region, sub-Saharan Africa, and Kurdish areas). [2] = coefficient differs significantly from the one observed for students from other ethnically minoritized groups.
$p < .001$ for all statistically significant differences.

Table S2

Results of Regression Analyses Predicting Discriminatory Climate (Based on a Different Grouping Procedure for Students with one Foreign-Born Parent)

	Model 1				
	ß	*SE*	*95% CI*		*p*
			LL	*UL*	
Minoritized student status (Ref.: minoritized students from stigmatized ethnic groups)					
Ethnic majority	-.09	.05	-.19	.00	.051
Students from other ethnically minoritized groups	-.07	.04	-.15	.02	.112
Gender	.08**	.03	.03	.12	.002
Age	.01	.03	-.05	.06	.749
Language spoken at home	.05	.03	-.01	.12	.093
SES[a]	-.01	.03	-.07	.04	.690
School track	-.13***	.03	-.18	-.08	<.001
R^2			.04**		.001

Note. Different from the analyses in the main body of the manuscript, an alternative grouping procedure for students with one foreign-born parent was used in these analyses. Specifically, these students were considered as either minoritized students from stigmatized ethnic groups or as students from other ethnically minoritized groups based on their parent's country of birth. ß = standardized regression coefficient. SE = standard error. CI = confidence interval. The group of "minoritized students from stigmatized ethnic groups" included minoritized students with heritage from Turkey, the SWANA region, sub-Saharan Africa, and Kurdish areas. The group of "students from other ethnically minoritized groups" comprised minoritized students from any other country. Gender is coded as 0 = girl, 1 = boy. Language spoken at home is coded as 0 = only or mostly German spoken at home, 1 = mostly a language other than German. The school track is coded as 0 = nonacademic track and 1 = academic track.
[a]SES = Socioeconomic status.
* $p < .05$; ** $p < .01$; *** $p < .001$.

Table S3

Test of Measurement Equivalence for Discriminatory Climate Across the Three Ethnic Groups

Model	X^2	*df*	*p*	*RMSEA*	*CFI*	*TLI*	*SRMR*	*AIC*	Comparison	ΔX^2	Δdf	*p*
configural	92.124	6	<.001	.165	.969	.906	.027	11942.871				
metric	97.399	12	<.001	.116	.969	.954	.034	11936.145	configural	5.275	6	.509
scalar	107.768	18	<.001	.097	.967	.967	.036	11934.514	metric	10.368	6	.110

Table S4

Test of Measurement Equivalence for Sense of School Belonging Across the Three Ethnic Groups

Model	X^2	*df*	*p*	*RMSEA*	*CFI*	*TLI*	*SRMR*	*AIC*	Comparison	ΔX^2	Δdf	*p*
configural	657.606	27	<.001	.175	.853	.755	.071	29270.151				
metric	717.318	37	<.001	.155	.842	.807	.085	29309.863	configural	59.712	10	<.001
scalar	756.362	47	<.001	.141	.835	.842	.096	29328.906	metric	39.044	10	<.001

Table S5

Test of Measurement Equivalence for Intrinsic Reading Motivation Across the Three Ethnic Groups

Model	X^2	*df*	*p*	*RMSEA*	*CFI*	*TLI*	*SRMR*	*AIC*	Comparison	ΔX^2	Δdf	*p*
configural	424.100	15	<.001	.170	.946	.892	.037	34618.681				
metric	444.645	23	<.001	.139	.944	.927	.045	34623.225	configural	20.544	8	.009
scalar	480.817	31	<.001	.124	.940	.942	.048	34643.397	metric	36.172	8	<.001

Table S6

Test of Measurement Equivalence for the Three Dimensions of Teaching Quality Across the Three Ethnic Groups

Model	X^2	*df*	*p*	*RMSEA*	*CFI*	*TLI*	*SRMR*	*AIC*	Comparison	ΔX^2	Δdf	*p*
configural	454.078	186	<.001	.039	.981	.977	.032	83690.407				
metric	467.427	206	<.001	.037	.982	.979	.034	83663.756	configural	13.350	20	.862
scalar	494.815	226	<.001	.036	.981	.981	.035	83651.143	metric	27.387	20	.125

Table S7

Pairwise Correlations and Descriptive Statistics of Variables at the Student Level

		(1)	(2)	(3)	(4)	(5)	(6)	(7)	(8)	(9)	(10)	(11)
1)	Discriminatory climate	-										
2)	Reading comprehension	-.32***	-									
3)	Intrinsic reading motivation	-.18***	.49***	-								
4)	Sense of school belonging	-.15***	.34***	.27***	-							
5)	Classroom management	-.16***	.25***	.36***	.38***	-						
6)	Individual student support	.02	-.20***	-.09***	-.02	.24***	-					
7)	Cognitive activation	<.01	-.22***	-.11***	-.04*	.30***	.72***	-				
8)	Age	.10***	-.35***	-.24***	-.17***	-.08***	.10***	.09***	-			
9)	Gender	.08***	-.15***	-.13***	-.05*	-.09***	.02	.05*	.10***	-		
10)	Language spoken at home	.11***	-.29***	-.08***	-.10***	-.06**	.10***	.09***	.22***	.01	-	
11)	SES [a]	-.11***	.38***	.31***	.23***	.13***	-.11***	-.14***	-.27**	-.04*	-.26**	-
	Mean/%	1.74	496.19	2.34	3.18	2.92	2.90	2.76	15.47	51.6%	17.8%	-0.06
	SD	0.62	98.70	0.87	0.57	0.72	0.84	0.69	0.65	-	-	0.99
	Min	1	188.06	1	1	1	1	1	13.67	1	1	-4.46
	Max	4	789.45	4	4	4	4	4	19.08	2	2	2.62

Note. Analyses are based on the first imputed dataset. N_{L1} = 2,947. [a]SES = Socioeconomic status. Gender is coded as 0 = girl and 1 = boy. Language spoken at home is coded as 0 = only or mostly German and 1 = mostly a language other than German.
* $p < .05$; ** $p < .01$; *** $p < .001$.

Table S8

Pairwise Correlations and Descriptive Statistics of Variables at the Classroom Level

		(1)	(2)	(3)	(4)	(5)	(6)	(7)	(8)
1)	Discriminatory climate	-							
2)	Classroom management	-.38***	-						
3)	Individual student support	.06	.25***	-					
4)	Cognitive activation	.06	.31***	.73***	-				
5)	Proportion of ethnically minoritized students	.34***	-.07	.21**	.26***	-			
6)	Proportion of minoritized students from stigmatized ethnic groups	.32***	-.09	.16*	.12	.70***	-		
7)	School track	-.48***	.23**	-.21**	-.28***	-.25***	-.19**	-	
8)	SES [a]	-.55***	.20**	-.26***	-.33***	-.47***	-.45***	.61***	-
	Mean/%	1.73	2.91	2.91	2.77	23.60%	7.51%	36.81%	-0.11
	SD	0.21	0.40	0.41	0.31	31.31%	12.25%	-	0.58
	Min	1.23	1.86	1.33	1.29	0%	0%	0	-1.90
	Max	2.30	3.70	4.00	3.60	100%	67.00%	1	1.10

Note. Analyses are based on the first imputed dataset. $N_{L2} = 201$. [a]SES = Socioeconomic status. The school track is coded as 0 = nonacademic track and 1 = academic track. The proportion of ethnically minoritized students refers to all ethnically minoritized students in class (i.e., minoritized students from stigmatized ethnic groups and minoritized students from other ethnic groups). The proportion of minoritized students from stigmatized ethnic groups refers to ethnically minoritized students with heritage from Turkey, the SWANA region, sub-Saharan Africa, and Kurdish areas.
* $p < .05$; ** $p < .01$; *** $p < .001$.

Table S9

Descriptive Statistics of Variables at the Student Level (by Student Group)

		Ethnic majority students		Minoritized students from stigmatized ethnic groups		Students from other ethnically minoritized groups	
		N = 2,304		*N* = 198		*N* = 445	
		M	*SD*	*M*	*SD*	*M*	*SD*
1)	Discriminatory climate	1.70 [1,2]	0.60	2.01 [2]	0.71	1.81	0.62
2)	Reading comprehension	509.71 [1,2]	93.44	422.63 [2]	90.17	458.91	104.79
3)	Intrinsic reading motivation [a]	--	--	--	--	--	--
4)	Sense of school belonging [a]	--	--	--	--	--	--
5)	Classroom management	2.92	0.72	2.85	0.74	2.93	0.69
6)	Individual student support	2.87 [1,2]	0.84	3.04	0.80	3.02	0.83
7)	Cognitive activation	2.72 [1,2]	0.68	2.92	0.65	2.90	0.69
8)	Age	15.40 [1,2]	0.57	15.82 [2]	0.85	15.68	0.79
9)	Gender	51.2%	--	55.1%	--	52.6%	--
10)	Language spoken at home	5.0% [1,2]	--	70.2% [2]	--	60.7%	--
11)	SES [b]	0.10 [1,2]	0.92	-0.95 [2]	1.13	-0.46	0.94
12)	School track	41.0% [1,2]	--	22.7%	--	29.7%	--

Note. Analyses are based on the first imputed dataset. N_{L1} = 2,947. [a]As scalar measurement invariance across groups was not given for this measure and means can, thus, not reasonably be compared across groups, no group-specific means are displayed here. [a]SES = Socioeconomic status. Gender is coded as 0 = girl and 1 = boy. Language spoken at home is coded as 0 = only or mostly German and 1 = mostly a language other than German. The school track is coded as 0 = nonacademic track and 1 = academic track.
[1] = coefficient differs significantly from the one observed for minoritized students from stigmatized ethnic groups (i.e., with heritage from Turkey, the SWANA region, sub-Saharan Africa, and Kurdish areas). [2] = coefficient differs significantly from the one observed for students from other ethnically minoritized groups.
$p < .001$ for all statistically significant differences.
